# miR-30b, Down-Regulated in Gastric Cancer, Promotes Apoptosis and Suppresses Tumor Growth by Targeting Plasminogen Activator Inhibitor-1

**DOI:** 10.1371/journal.pone.0106049

**Published:** 2014-08-29

**Authors:** En-Dong Zhu, Na Li, Bo-Sheng Li, Wei Li, Wei-Jun Zhang, Xu-Hu Mao, Gang Guo, Quan-Ming Zou, Bin Xiao

**Affiliations:** 1 National Engineering Research Center of Immunological Products, Department of Microbiology and Biochemical Pharmacy, College of Pharmacy, Third Military Medical University, Chongqing, P.R. China; 2 2011 Collaborative Innovation Center of Tianjin for Medical Epigenetics, Key Laboratory of Hormones and Development (Ministry of Health), Metabolic Diseases Hospital & Tianjin Institute of Endocrinology, Tianjin Medical University, Tianjin, P.R. China; 3 Department of Pharmacy, Southwest Hospital, Third Military Medical University, Chongqing, P.R. China; Federico II University of Naples, Italy, Italy

## Abstract

**Background:**

Gastric cancer is one of the most common malignant diseases worldwide. Emerging evidence has shown that microRNAs (miRNAs) are associated with tumor development and progression. Our previous studies have revealed that *H. pylori* infection was able to induce the altered expression of miR-30b in gastric epithelial cells. However, little is known about the potential role of miR-30b in gastric cancer.

**Methods:**

We analyzed the expression of miR-30b in gastric cancer cell lines and human gastric cancer tissues. We examined the effect of miR-30b mimics on the apoptosis of gastric cancer cells in vitro by flow cytometry (FCM) and caspase-3/7 activity assays. Nude mouse xenograft model was used to determine whether miR-30b is involved in tumorigenesis of gastric cancer. The target of miR-30b was identified by bioinformatics analysis, luciferase assay and Western blot. Finally, we performed the correlation analysis between miR-30b and its target expression in gastric cancer.

**Results:**

miR-30b was significantly down-regulated in gastric cancer cells and human gastric cancer tissues. Enforced expression of miR-30b promoted the apoptosis of gastric cancer cells in vitro, and miR-30b could significantly inhibit tumorigenicity of gastric cancer by increasing the apoptosis proportion of cancer cells in vivo. Moreover, plasminogen activator inhibitor-1 (PAI-1) was identified as the potential target of miR-30b, and miR-30b level was inversely correlated with PAI-1 expression in gastric cancer. In addition, silencing of PAI-1 was able to phenocopy the effect of miR-30b overexpression on apoptosis regulation of cancer cells, and overexpression of PAI-1 could suppressed the effect of promoting cell apoptosis by miR-30b, indicating PAI-1 is potentially involved in miR-30b-induced apoptosis on cancer cells.

**Conclusion:**

miR-30b may function as a novel tumor suppressor gene in gastric cancer by targeting PAI-1 and regulating the apoptosis of cancer cells. miR-30b could serve as a potential biomarker and therapeutic target against gastric cancer.

## Introduction

Gastric cancer causes about 738,000 deaths worldwide per year, and it has been recognized as the third leading cause of cancer-related death in men [1]. Early diagnosis and treatment have led to excellent expectations for long-term survival and good prognosis, whereas the outlook for patients with advanced gastric cancer remains poor. Like other cancers, the development of gastric cancer is thought to be multifactorial. *Helicobacter pylori(H. pylori)* infection has been recognized to be an important trigger of gastric cancer [2]. Although many genetic and epigenetic changes have been reported in gastric cancer, the molecular mechanism underlying the development of gastric cancer remains unclear.

microRNAs (miRNAs) are small non-coding RNAs that posttranscriptionally regulate gene expression. Mature miRNAs can specifically bind to 3′ UTRs of target cellular mRNA in turn triggering mRNA degradation or inhibition of translation [3], [4]. miRNAs act as key regulators in a wide variety of biological processes, including development, cell differentiation, apoptosis, metabolism, and signal transduction [5], [6]. It has been demonstrated that 50% of miRNAs are frequently located at cancer-associated genomic regions or in fragile sites [7]. Growing evidence has shown that aberrant miRNAs expression correlates with various human cancers and indicates that miRNAs can function as oncogenes or tumour-suppressor genes [8]–[10]. Recently, a substantial number of deregulated miRNAs including miR-106b-25 cluster, miR-21, miR-218, miR-7, and miR-335 have been identified as modulators of cell growth, apoptosis, migration, or invasion in gastric cancer development [11]–[15]. These findings suggest the miRNAs may play a crucial role in the pathogenesis of gastric cancer.

Our previous studies have revealed that *H. pylori* infection was able to induce the altered expression of miRNAs in gastric epithelial cells including miR-155, miR-146a and miR-30b, miRNAs may function as novel negative regulators to fine-tune *H. pylori*-induced inflammation [16], [17]. Moreover, we demonstrated that miR-146a may play potential role in development of gastric cancer by modulating the proliferation and apoptosis of gastric cancer cells [18]. Notably, we also found miR-30b could regulate the autophagy process during *H. pylori* persist infection, thereby contributing to the persistence of *H. pylori* infections [19]. However the role of miR-30b in gastric cancer is still largely unknown.

Plasminogen activator inhibitor 1 (PAI-1) is the main serine protease inhibitor of tissue-type (t-PA) and urokinase-type (u-PA) plasminogen activator and therefore plays an important role in the plasminogen-plasmin system [20]. Previous studies have illustrated PAI-1 is a poor prognostic factor in several common tumors, and is associated with cancer invasion and metastasis [21]. Recently, many groups also have found that PAI-1 may promote tumor growth through inhibition of cell apoptosis. For instance, addition of a stable wild-type PAI-1 to the human prostate cancer cell line PC-3, the human promyelocytic leukaemia cell line HL-60 and even non-tumoural cells, resulted in a significant inhibition of apoptosis [22]. When injected subcutaneously into nude mice with PAI-1 overexpressing murine fibrosarcoma cells, the tumors were rapidly established, while PAI-1 deficient cells had a suppressed tumorigenicity [23]. Another report showed that, genetic and pharmacological inhibition of PAI-1 in human tumor cell lines (HT-1080, A549, HCT-116, and MDA-MB-231) increased spontaneous apoptosis, and interestingly, implanted PAI-1 knockdown HT-1080 cells to PAI-1 knockout mice resulted in decreased tumorigenesis and prolonged survival compared with control mice [24]. These studies provide important evidence that PAI-1 exerts a protective role against tumor cell apoptosis.

In current study, we found that miR-30b was significantly down-regulated in gastric cancer cells and tumor tissues. Ectopic expression of miR-30b could promote the apoptosis of gastric cancer cell in vitro, and miR-30b could significantly inhibit tumorigenicity of gastric cancers in nude mouse xenograft model. Moreover, PAI-1 was identified as the potential targets of miR-30b, and miR-30b may induce apoptosis and suppress tumor growth by repressing the expression of PAI-1. Our findings suggest that miR-30b may function as a novel tumor suppressor gene in gastric cancer and can be a potential therapy target for gastric cancer.

## Materials and Methods

### Ethics Statement

All of the animal experiments were approved by the Animal Ethical and Experimental Committee of the Third Military Medical University. All animal surgery was performed under sodium pentobarbital anesthesia, and all efforts were made to minimize suffering. The study involving tissue specimens was approved by the Ethics Review Board at Third Military Medical University, and written informed consent was obtained from all patients before participation.

### Tissue specimens

In total, gastric cancer tissues and adjacent non-tumor tissues were obtained from patients with primary gastric cancer undergoing radical gastrectomy at Southwest Hospital, Third Military Medical University, China, and the clinicopathologic characteristics of gastric cancer patients are summarized in Table 1. After surgical removal, the tissues were frozen immediately in liquid nitrogen and stored at −80°C. The study was approved by the ethics review board at Third Military Medical University, and informed consent was obtained from all patients before participation.

**Table 1:** Summary of clinicopathological parameters of patients with gastric cancer.

	Clinicopathological parameters	Number of cases (%)
**Gender**	Male	13 (62)
	Female	8 (38)
**Age**	≤60	10 (48)
	>60	11 (52)
**Diameter (cm)**	≤5	9 (43)
	>5	12 (57)
**Local invasion**	T1, T2	9 (43)
	T3, T4	12 (57)
**Lymph node metastasis**	Negative	12 (57)
	Positive	9 (43)
**TNM stage**	I, II	11 (52)
	III, IV	10 (48)

### Cell lines and cell culture

All cell lines used in this study were obtained from Shanghai Type Culture Collection of Chinese Academy of Sciences (Shanghai, China), the human gastric cancer cell lines AGS was cultured in Ham's F12 (Hyclone) medium with 10% fetal bovine serum (FBS), other gastric cancer cell lines MKN45, HGC-27, BGC-823, SGC-7901 and human embryonic kidney (HEK) 293 cells were cultured in DMEM (Hyclone) with 10% FBS, they were all maintained in a humidified incubator containing 5% CO_2_ at 37°C as previously described.

### Quantitative RT-PCR

Total RNA was extracted with Trizol reagent (Invitrogen), followed by a reverse transcription using TaqMan miRNA assays (Ambion), with U6 small nuclear RNA as an internal normalized reference. qRT-PCR reactions were performed using the following parameters: 95°C for 2 min followed by 40 cycles of 95°C for 15 s and 60°C for 30 s. qRT-PCR analyses for the mRNA of PAI-1 were performed by using PrimeScript RT-PCR kits (Takara), as the following parameters: 95°C for 30 s followed by 40 cycles of 95°C for 5 s, 60°C for 5 s and 72°C for 30 s. The mRNA level of β-actin was used as an internal control. The sequences of primers used are shown in Table 2.

**Table 2:** Sequences of oligonucleotides used in the paper.

Name	Sequences
miR-30b-mimics-sense	5′-UGUAAACAUCCUACACUCAGCU-3′
miR-30b-mimics-antisense	5′-CUGAGUGUAGGAUGUUUACAUU-3′
miR-Control-sense	5′-UUCUCCGAACGUGUCACGUTT-3′
miR-Control-antisense	5′-ACGUGACACGUUCGGAGAATT-3′
PAI-1-sense	5′-CTAGTGGGCCCATTTTGGAGTGTAGGTGACTTGTTTACTCATTGAAGCAGATTTCTGCA-3′
PAI-1-antisense	3′-ACCCGGGTAAAACCTCACATCCACTGAACAAATGAGTAACTTCGTCTAAAGACGTTCGA-5′
PAI-1-mut-sense	5′- CTAGTGGGCCCATTTTGGAGTGTAGGTGACTCGAAGTATCATTGAAGCAGATTTCTGCA-3′
PAI-1-mut-antisense	3′- ACCCGGGTAAAACCTCACATCCACTGAGCTTCATAGTAACTTCGTCTAAAGACGTTCGA-5′
PAI-1-F	5′-CAGACCAAGAGCCTCTCCAC-3′
PAI-1-R	5′-ATCACTTGGCCCATGAAAAG-3′
PAI-1 siRNA sense	5′-CCAGAUUCAUCAUCAAUGATT-3′
PAI-1 siRNA antisense	5′-UCAUUGAUGAUGAAUCUGGTT-3′
NC control sense	5′-UUCUCCGAACGUGUCACGUTT-3′
NC control antisense	5′-ACGUGACACGUUCGGAGAATT-3′

### Northern blot

Small-sized RNAs was isolated using the mirVana RNA Isolation kit (Ambion). Three pairs of clinical samples were randomly chosen from the 21 patients for subsequent Northern blot analysis. Northern blot was performed according the standard procedure as descried previously [17]. The DNA oligonucleotide antisense probes used to detect miR-30b and U6 snRNA were as follows: miR-30b (5′- AGCTGAGTGTAGGATGTTTACA-3′) and U6 (5′-ATATGGAACGCTTCACGAATT-3′).

### Cell transfection

miR-30b mimics, scrambled miR-control, chemically modified miR-30b duplex (agomir), chemically modified scrambled miR-control, PAI-1 siRNA, or siRNA negative control were purchased from GenePharma (Shanghai GenePharma Co. Ltd., China). PAI-1 (Serpine1) Human cDNA ORF Clone was purchased from Origene (Origene Technologies, Beijing, China). Transfections were performed using Lipofectamine 2000 (Invitrogen) according to the manufacturer's protocol.

### Cell apoptosis assay by FCM

SGC-7901 or HGC-27 cells were seeded in 12-well plate at a suitable density and grown to 30% confluency after 24 h. Then cells were transfected with miR-30b mimics or miR-control, and the medium was replaced with serum-free DMEM for 48 h. For co-transfection experiment with miR-30b and PAI-1 expressing vector, SGC-7901 cells were transfected with miR-control or miR-30b mimics, and then with PAI-1 Human cDNA ORF Clone vector (indicated as PAI-1) or empty vector 24 h later. 24 h after vector transfection, the medium was replaced with serum-free DMEM for another 24 h. Finally, the cells were applied to apoptosis analysis. For FCM analysis, an Annexin V-FITC Apoptosis Detection Kit I (BD Pharmingen) was used, then analysed by flow cytometry (BD FACSCantoTM II).

### Caspase-Glo 3/7 Assay

The activity of caspase-3/7 was detected using the Caspase-Glo 3/7 Assay (Promega). Add 100 µl of Caspase-Glo 3/7 reagent to white-walled 96-well plate, gently mix contents of wells, and then incubate the wells at room temperature for 30 min. The luminescence was detected using the GloMax-96 Microplate Luminometer (Promega).

### Tumorigenicity assays in nude mice

The mice used in this experiment were maintained under specific pathogen-free conditions. Viable miR-30b mimics- and miR-control-transfected SGC-7901 cells (1×10^6^) were suspended in 100 µl PBS and then injected subcutaneously into either side of the posterior flank of the same female BALB/c athymic nude mouse at 4 to 6 weeks of age as described previously [25]. Tumor growth and the condition including the overall health and behavior of the mice were examined every three days for 4 weeks. The tumor beared mice did not exhibit signs of pain or distress such as weight loss or behavioral changes, so all mice were sacrificed by CO_2_ asphyxiation on day 28 post-injection. Tumor volume (V) was monitored by measuring the length (L) and width (W) with calipers and calculated with the formula (L×W^2^) ×0.5.

### TUNEL staining

The tumors were harvested at 26 days, and fixed in 10% formaldehyde solution, then embedded in paraffin. Sections (5 µm thick) that had been deparaffinized and rehydrated were stained with hematoxylin and eosin. Apoptotic tumor cells were stained by in situ terminal deoxynucleotidyltransferase-mediated dUTP nick end labeling (TUNEL) method using an in situ cell death detection kit (POD; Roche Diagnostics Co.). Negative control was subjected to the same staining for TUNEL without TdT. Images were collected by using microscope with ×200 magnification.

### Construction of plasmids

The construction of various luciferase report vectors for miR-30b target was performed as previously described [16], [26], and the construct containing mutant seed region was generated as a control. The sequences of oligonucleotides used are shown in Table 2.

### Luciferase assay and GFP repression experiments

HEK-293 cells were seeded in 96-well plate at 5,000 cells per well the day before transfection. The cells were transfected with each firefly luciferase reporter vector, Renilla luciferase control vector, pRL-TK (Promega), and miR-30b mimics or miR-control (GenePharma). The luciferase assay was performed by using the dual luciferase reporter assay system (Promega).

For GFP repression experiments, HEK-293 cells were seeded in 12-well plate at 1×10^5^ per well the day before transfection and then were cotransfected with the miR-30b mimics or miR-control with various GFP reporter vectors. The cells were subjected to flow cytometric analysis, and data were analyzed by using CellQuest Pro software.

### Western blot

Treated cells were washed with ice-cold PBS and then lysed by a cell lysis buffer (Pierce). After centrifugation at 12,000 rpm for 15 min at 4°C, the protein concentration was measured by BCA protein assay kit (Pierce). Cell protein lysates were separated in 12% SDS denatured polyacrylamide gel and then transferred onto a polyvinylidene difluoride membrane. The membranes were blocked with 5% nonfat dry milk in Tris-buffered saline, pH 7.4, containing 0.05% Tween 20, and were incubated with primary antibodies for PAI-1(1∶1000, Abcam) and β-actin (1∶1000, Santa Cruz) at 4°C overnight respectively. Membranes were washed and incubated with horseradish peroxidase-conjugated secondary antibodies (1∶5000, Santa Cruz) according to the manufacturer's instructions. The protein of interest was visualized using ECL Western blotting substrate (Pierce) and Chemidoc XRS Gel Documentation System (BioRad).

### Statistical analysis

The results are expressed as mean ± standard deviation (S.D.) from at least three independent experiments. Student's t test was applied to analyze the differences between groups. The relationship between the miR-30b level and PAI-1expression was analyzed using Pearson's correlation. Statistical analysis was performed with SPSS software (version 17). Statistical differences were declared significant at *P*<0.05 level. Statistically significant data are indicated by asterisks (*P*<0.05 (*), *P*<0.01(**).

## Results

### Decreased miR-30b expression in human gastric cancer cell lines and GC tissue samples

**Figure 1:**
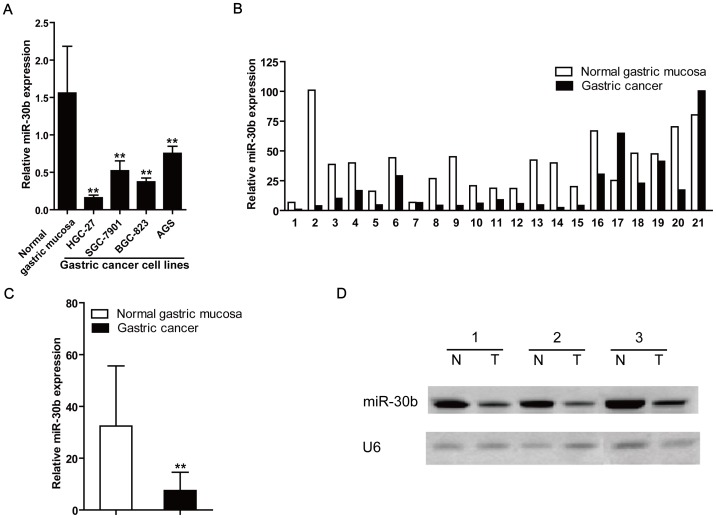
Decreased miR-30b expression in human gastric cancer cell lines and tumor tissues. (**A**) Comparison of expression level of miR-30b between normal gastric mucosa tissue samples and gastric cancer cell lines HGC-27, SGC-7901, BGC-823 and AGS. **P<0.01, compared with normal tissues. (**B**) Relative expression of miR-30b in 21 gastric cancer tissues compared with matched adjacent non-tumor gastric tissues. (**C**) Comparison of the average expression of miR-30b in two groups of B. Data represent means±S.D. **P<0.01, compared with normal gastric mucosa. (**D**) Analysis of miR-30b expression by using Northern blotting. Total RNA was extracted from three paired gastric cancer (T) and adjacent non-cancerous gastric tissues (N), which were randomly chosen from all patients. RNAs were hybridized sequentially with miR-30b and U6 probe, and U6 was used as a control for RNA loading.

To determine the role of miR-30b in the pathogenesis of gastric cancer, we analyzed the miR-30b levels in various gastric cancer cells, including HGC-27, AGS, BGC-823 and SGC-7901. As shown in Figure 1A, the expression of miR-30b was significantly down-regulated in four cell lines compared with a pool of five non-tumor gastric tissues (*P*<0.01).

The expression of miR-30b was further examined in 21 paired GC and adjacent non-tumor gastric tissues through TaqMan quantitative real-time PCR (qRT-PCR). As shown in the Figure 1B, the decrease of miR-30b was found in 18 of 21 patients (85.7%) compared with the corresponding non-tumor tissues. Furthermore, the scatter diagram showed that miR-30b was significantly down-regulated in gastric cancer samples versus normal gastric mucosa, with an average 6.28-fold decrease (*P* = 0.002) (Figure 1C). To validate the expression data acquired from qRT-PCR, 3 of all 21 pairs samples were randomly chose to determine the level of miR-30b using Northern bolt. We also found the consistent decreased expression of miR-30b in gastric cancer compared with non-tumor gastric tissues (Figure 1D). These results suggest that the miR-30b expression is frequently down-regulated in gastric cancer and maybe involved in the development of gastric cancer.

### Overexpression of miR-30b increases the apoptosis of gastric cancer cells

**Figure 2:**
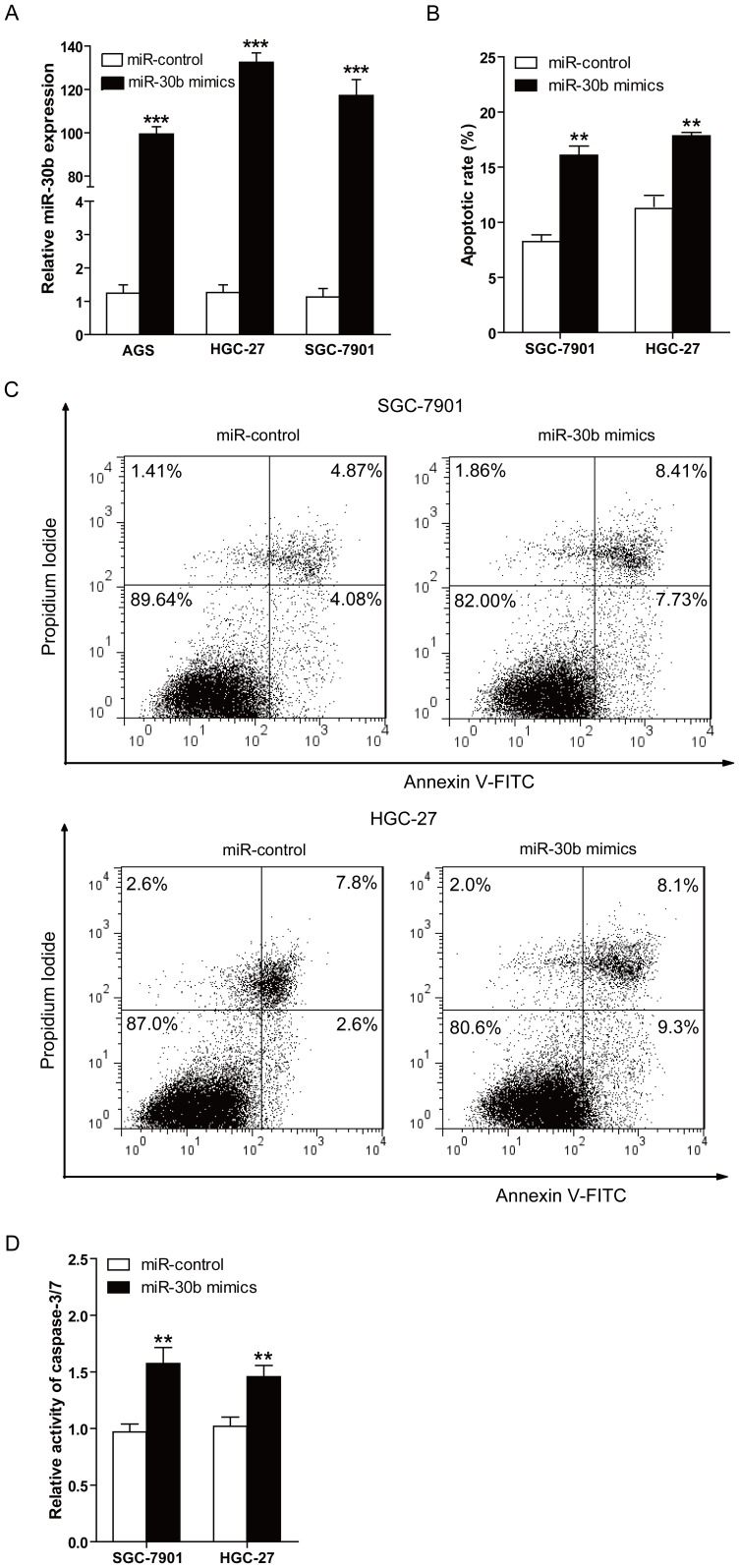
Overexpression of miR-30b increases the apoptosis of gastric cancer cells. (**A**) Relative expression of miR-30b in AGS, HGC-27, and SGC-7901 cells transfected with miR-30b mimics or miR-control for 48 h. Data represent means±S.D. from three independent experiments, ***P<0.001. (**B**) The effect of miR-30b on apoptosis was examined by FCM analysis. SGC-7901 or HGC-27 cells were transfected with miR-30b mimics or miR-control, and then the medium was replaced with serum-free DMEM for 48 h, cells were analyzed for apoptotic rate after staining with Annexin V-FITC and PI. Data represent means±S.D. from four independent experiments, **P<0.01. (**C**) One representative FCM analysis in B is shown. (**D**) SGC-7901 or HGC-27 cells were transfected with miR-30b mimics or miR-control for 48 h. The activity of caspase-3 and caspase-7 was detected using the Caspase-Glo 3/7 Assay. Data are mean ± S.D. of 6 duplications from four independent experiments, **P<0.01.

One of the hallmark of cancer is its ability to evade apoptosis [27], so we examined the effect of miR-30b on gastric cancer cells apoptosis. SGC-7901 or HGC-27 cells were transfected with miR-30b mimics or scrambled miR-control, and the validity of miR-30b ectopic expression was confirmed by qRT-PCR (Figure 2A). Then the effect of miR-30b on apoptosis of SGC-7901 or HGC-27 cells was evaluated by flow cytometry (FCM) analysis and caspase-3/7 activity assays. As shown in Figure 2B and 2C, we found the proportion of apoptotic cells was significantly increased in SGC-7901 or HGC-27 cells transfected with miR-30b mimics compared with miR-control-transfected cells (16.08% *versus* 8.25% to SGC-7901 cells, and 17.83% *versus* 10.87% to HGC-27 cells). Furthermore, significant increase in caspase-3/7 activity was found in SGC-7901 or HGC-27 cells transfected with miR-30b compared with miR-control transfectants (*P*<0.01, Figure 2D). Above results reveal that overexpression of miR-30b can promote the apoptosis of gastric cancer in vitro.

### miR-30b suppresses tumorigenicity and promotes cell apoptosis in vivo

**Figure 3:**
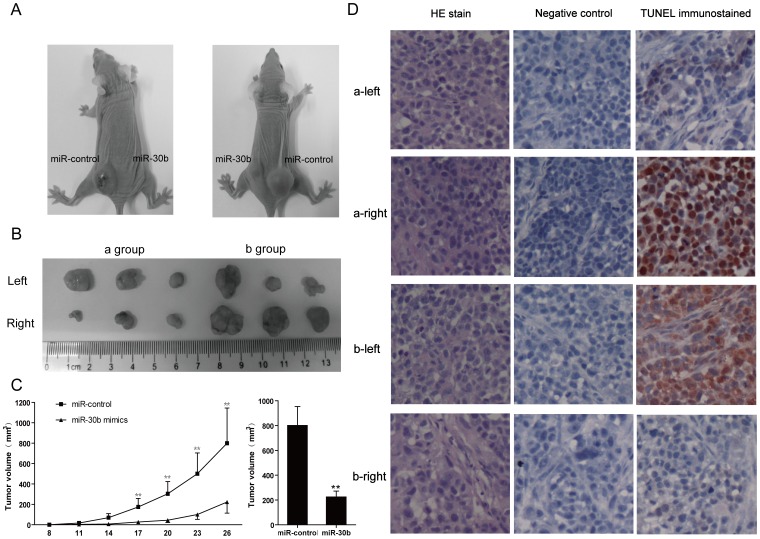
miR-30b suppresses tumorigenicity and promotes cell apoptosis in vivo. (**A**) Effect ofmiR-30b on tumor formation in vivo xenograft mode. miR-control- and miR-30b-transfected SGC-7901 (1×10^6^) were suspended in 100 µl PBS and then injected subcutaneously into either side of the posterior flank of the same female BALB/c athymic nude mouse. Two representative tumor-bearing mice after 4 weeks inoculation were shown. (**B**) The significant differences of tumor volume between miR-control group and miR-30b group from 6 mice. (**C**) The tumor volume was measured every three days after injection of SGC-7901 cells with treatment as described in **A**, and the tumor growth curve was shown. The difference of tumor size between miR-control group and miR-30b group was statistically significant, **P<0.01. (**D**) The tumors were harvested at 26 days from two sides of the posterior flank of the same mouse, apoptosis in situ were measured by TUNEL. All data above are representative at least three independent experiments.

To further determine whether miR-30b is involved in tumorigenesis of gastric cancer, nude mouse xenograft model was used. Because we found that miR-30b played a more significant role in promoting SGC-7901 cells apoptosis than HGC-27 cells in vitro, we explore the role of miR-30b in tumorigenesis of gastric cancer using SGC-7901 cells. miR-control- and miR-30b-tranfected SGC-7901 cells were injected subcutaneously into either posterior flank of the same nude mice. The mice were followed for observation of xenograft growth for 4 weeks. It was found that introduction of miR-30b into SGC-7901 cells led to a significant reduction in the size of tumor volume (Figure 3A and 3B), and tumors derived from miR-30b treated SGC-7901 cells grew slower compared with the control group during the whole tumor growth period (Figure 3C, left panel). When tumors were harvested, the average volume of tumors derived from the miR-30b mimics group was only 27.87% of that in the miR-control group (Figure 3C, right panel, *P*<0.01). The tumors were harvested at 26 days from two sides of the posterior flank of the same mouse, apoptosis in situ were measured by TUNEL. As shown in Figure 3D, tumor sections from miR-30b mimics treated xenografts exhibited significantly increase in TUNEL-positive cells. These results strongly suggested that introduction of miR-30b could inhibit gastric cancer growth by promoting apoptosis of cancer cells.

### PAI-1 is a candidate target gene of miR-30b

**Figure 4:**
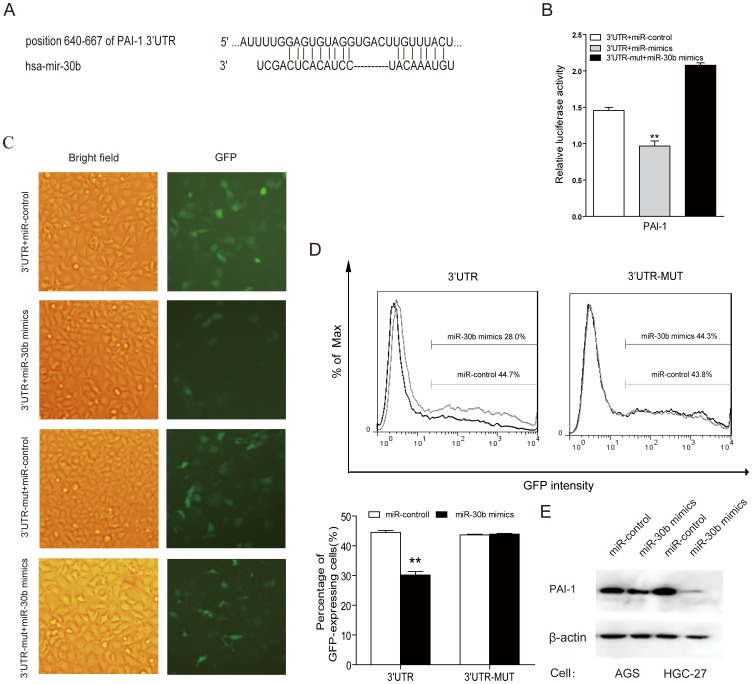
Identification of PAI-1 as a potential target of miR-30b. (**A**) Sequence alignment of miR-30b and its target sites in 3′-UTRs of PAI-1. (**B**) HEK293 cells were transiently cotransfected with luciferase report vectors, and either miR-30b mimics or miR-control. Luciferase activities were normalized to the activity of Renilla luciferase. (**C and D**) HEK-293 cells were transfected with the GFP report vector or mutant vector, and either miR-30b mimics or miR-control. GFP fluorescence was monitored by fluorescence microscope and FCM analysis. (**E**) Western blots analysis for PAI-1 of AGS and HGC-27 cells transfected with miR-30b mimics or miR-control. All data above are representative of at least three independent experiments, ** *P*<0.01.

To further assess the function of miR-30b, it is important to determine which host mRNAs are being regulated by miR-30b. In our previous study, we examined the mRNA expression profile in five pairs of primary tumor tissues of gastric cancer patients and matched non-tumor tissues using microarray. Totally, we found 303 upregulated genes (fold change >2, *P*<0.05) in gastric cancer tissues compared with non-tumor tissues (data not shown). Considering the downregulated expression of miR-30b in gastric cancer, we focused on the list of genes showing increased expressions and selected the top 30 increased genes in gastric cancer, then determined if those genes were potential targets of miR-30b by using the prediction algorithm, TargetScan. Interestingly, we found that PAI-1 gene which was upregulated in microarray (3.83 fold, *P* = 0.04) might be a probable target gene of miR-30b (Figure 4A). To directly address whether miR-30b binds to the 3′-UTR of the target mRNAs, we constructed the luciferase report vectors that contain the putative miR-30b binding sites within 3′-UTR. As shown in Figure 4B, we observed a marked reduction in luciferase activity (*P*<0.01) after contransfection of luciferase report vectors and miR-30b mimics. In contrast, no change of luciferase was observed in cells transfected with the mutant 3′-UTR constructs. This result was subsequently confirmed by GFP repression experiments. As shown in Figure 4C and 4D, GFP fluorescence was significantly reduced in cells transfected with GFP report vectors containing binding sites and miR-30b mimics, whereas there was no change of GFP fluorescence in cells transfected with mutant vector and miR-30b mimics. Furthermore, overexpression of miR-30b in AGS and HGC-27 cells resulted in the down-regulation of the protein levels of PAI-1 (Figure 4E). Taken together, above data suggest that PAI-1 is a potential target of miR-30b, and miR-30b might down-regulate the target protein.

### Inverse correlation between mir-30b and PAI-1 expression in gastric cancer tissues and cancer cell lines

**Figure 5:**
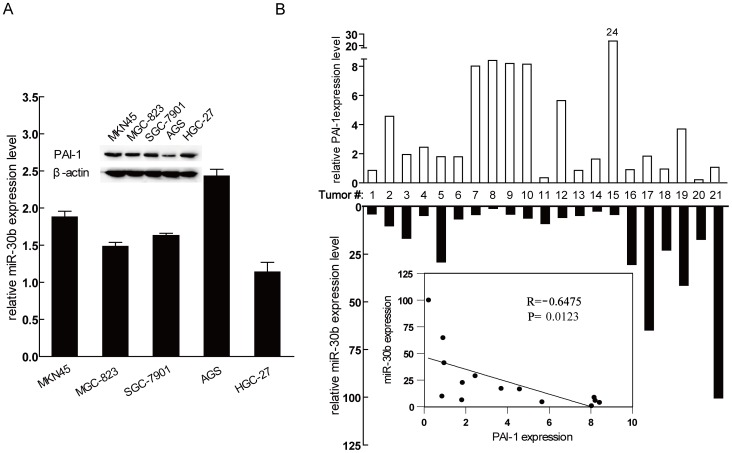
Inverse correlation between miR-30b and PAI-1 expression in gastric cancer cell lines and human tumor tissues. (**A**) The expression of mir-30b and PAI-1 in gastric cancer cell lines MKN45, MGC-823, SGC-7901, AGS and HGC-27. Top, western blot analysis of PAI-1 protein levels; bottom, qRT-PCR analysis of miR-30b levels. Data represent means±S.D. from three independent experiments. (**B**) miR-30b and PAI-1 mRNA levels in gastric cancer tissues were analyzed by qRT-PCR. The inset graph indicated a statistically significant inverse correlation (*R* = −0.6475, *P* = 0.0123). U6 and β-actin served as internal normalized references for miR-30b and PAI-1 mRNA, respectively.

Considering that miR-30b is down-regulated in gastric cancer, and it has been reported that PAI-1 protein in gastric cancer tissues is dramatically higher than in the non-tumor tissues [28], we performed the correlation analysis between miR-30b and PAI-1 expression in gastric cancer cell lines and gastric cancer tissues. As shown in Figure 5A top, PAI-1 protein level was the lowest in AGS cell among the five gastric cell lines. In contrast, miR-30b level was the highest in AGS, compared with other cell lines (Figure 5A bottom). Subsequently, we examined PAI-1 and miR-30b expression in 21 sets of gastric cancer and adjacent non-tumor tissues. We found that 81% (17/21) of gastric cancers displayed higher PAI-1 mRNA compared with normal tissues. In contrast, miR-30b was commonly down-regulated in 18 of 21 tumors. Using Pearson's correlation analysis, we observed a significant inverse correlation between miR-30b and PAI-1 mRNA (Figure 5B, *R* = −0.6475, *P* = 0.0123). Above results suggest that miR-30b expression is inversely correlated with PAI-1 expression in gastric cancer, and enhanced PAI-1 expression in gastric cancer could be a result of reduced miR-30b expression.

### PAI-1 is potentially involved in miR-30b-induced apoptosis

**Figure 6:**
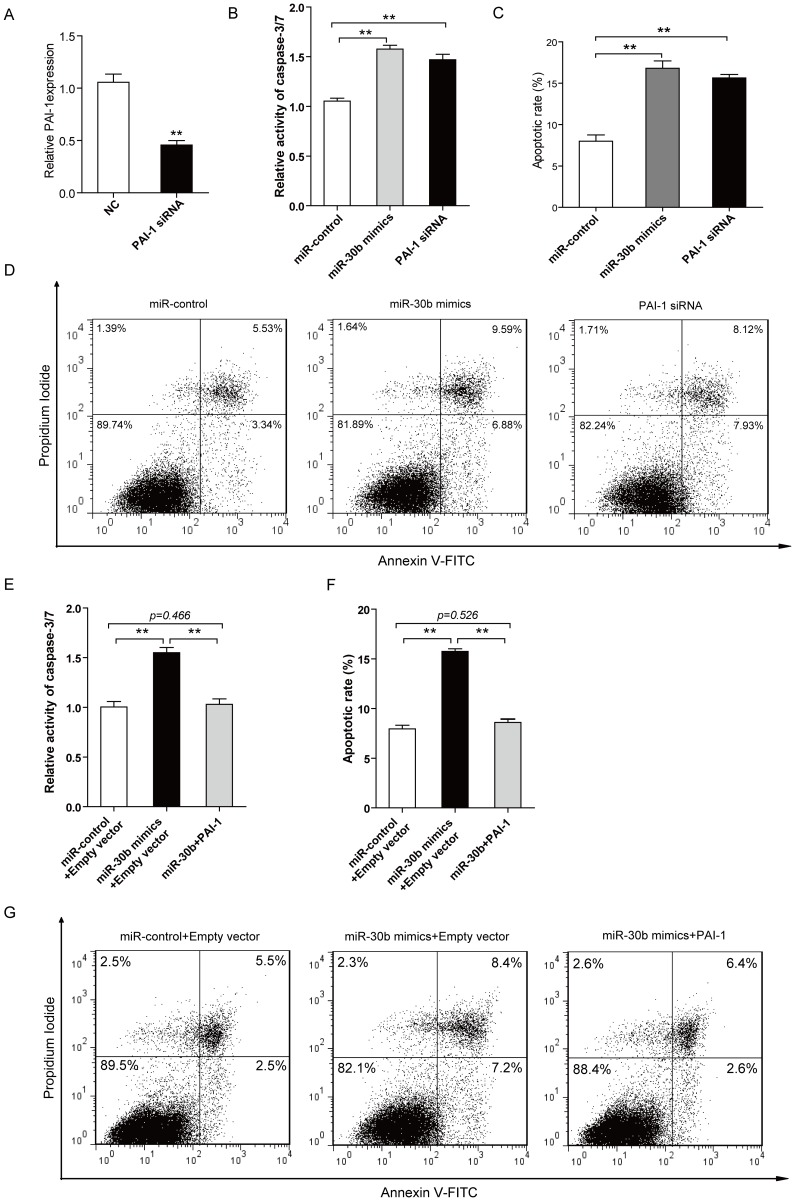
PAI-1 is involved in miR-30b-regulated apoptosis. (**A**) PAI-1 siRNA inhibits the expression of PAI-1 efficiently at mRNA levels. RT-PCR was used to monitor the expression of PAI-1 48 h after transfection with siRNA for PAI-1 or negative control RNA, β-actin served as an internal control. Data represent means±S.D. from three independent experiments, ** *P*<0.01. (**B**) SGC-7901 cells were transfected with miR-control, miR-30b or PAI-1 siRNA, and the medium was replaced with serum-free DMEM for 48 h, the activity of caspase-3 and caspase-7 was detected using the Caspase-Glo 3/7 Assay. Data are mean ± S.D. of 6 duplications from three independent experiments, **P<0.01. (**C**) SGC-7901 cells were treated as (**B**), and then cells were analyzed for apoptotic rate after staining with Annexin V-FITC and PI. Data represent means±S.D. from three independent experiments, ** *P*<0.01. (**D**) One representative FCM analysis in (**C**) was shown. (**E**) SGC-7901 cells were first transfected with miR-control or miR-30b mimics, and then with PAI-1 Human cDNA ORF Clone vector (indicated as PAI-1) or empty vector 24 h later. 24 h after vector transfection, the medium was replaced with serum-free DMEM for another 24 h, finally the activity of caspase-3 and caspase-7 was detected using the Caspase-Glo 3/7 Assay. Data are mean ± S.D. of 6 duplications from three independent experiments, **P<0.01. (**F**) SGC-7901 cells were treated as (**E**), finally the cells were analyzed for apoptotic rate after staining with Annexin V-FITC and PI. Data represent means±S.D. from three independent experiments, **P<0.01. (**G**) One representative stain in (**F**) is shown.

To further investigate whether PAI-1 is involved in miR-30b-promoted apoptosis, we first tested if the silencing of PAI-1 expression may have the similar apoptosis-promoting effect as miR-30b overexpression. SGC-7901 cells were transfected with PAI-1 siRNA or negative control RNA (NC) for 48 h, as shown in Figure 6A, the mRNA levels of PAI-1 can be significantly reduced by its siRNA. Subsequently, PAI-1 siRNA displayed obvious increases in the apoptotic rate in SGC-7901 cells (Figure 6B, 6C), compared with miR-control. Notably, PAI-1 siRNA was able to phenocopy the effect of miR-30b overexpression on apoptosis regulation of cancer cell (Figure 6B, 6C). One representative example of dot plot was shown in Figure 6D. Next, we also examined whether PAI-1 could abrogate the effect of promoting apoptosis by miR-30b. SGC-7901 cells were transfected with miR-control or miR-30b mimics for 24 h, and followed by transfection with PAI-1 Human cDNA ORF Clone vector (indicated as PAI-1) or empty vector. As shown in Figure 6E-G, the overexpressing of PAI-1 resulted in obvious suppression on the effect of miR-30b-induced apoptosis. Taken together, our results suggest that PAI-1 is potentially involved in miR-30b-promoted apoptosis.

## Discussion

Although deregulation of miRNAs has been observed in gastric cancer tissues and cell lines, the exact molecular mechanism by which miRNAs modulate the process of tumorigenesis is not yet fully elucidated. To date, a series of miRNAs has been reported to function as oncogene in the development of gastric cancer [29], [30], however miRNAs acting as tumor suppressor genes need to be further investigated.

Here, we showed miR-30b was down-regulated in gastric cancer cells and tumor tissues. Our data also indicated that overxepression of miR-30b could improve the apoptosis of gastric cancer cells in vitro and in vivo, and miR-30b was able to suppress tumor growth of gastric cancer in vivo. We further characterized PAI-1 as a potential target of miR-30b, and the expression of miR-30b was inversely correlated with PAI-1 expression in gastric cancer. In addition, PAI-1 is potentially involved in miR-30b-induced apoptosis. Above findings suggest miR-30b may act as a novel tumor suppressor by regulating the apoptosis of cancer cell in gastric cancer.

miR-30b is one of the miR-30 family which is associated with the development of many types of cancers. However, the role of miR-30b in tumorigenesis is controversial. miR-30b was reported to be down-regulated in prostate cancer [31], invasive bladder tumor [32], anaplastic thyroid cancer [33], esophageal cancer[34], and lung cancer[35], whereas enhanced expression of miR-30b was identified in medulloblastoma [36] and malignant mesothelioma [37]. In addition, miR-30b was identified as one of independent predictors for recurrence-free survivals of hepatocellular carcinoma [38]. Since miR-30b was either up-regulated or reduced in different cancers, we could draw a conclusion that miR-30b may play different roles as an oncogene or a tumor suppressor gene in various cancers.

In the current study, we found that miR-30b expression was significantly decreased in gastric cancer tissues and cell lines compared with normal gastric tissues. Consistent with our finding, miR-30b was found to be down-expressed by microRNA array from 184 gastric cancers and 169 non-tumor mucosae [39], and Qiao et al found that miR-30b was down-expressed in gastric cancer tissue and gastric cancer cell lines, AGS and BGC-823 cells [40]. Therefore, the loss of miR-30b expression may be associated with the pathogenesis and progression of gastric cancer.

Apoptosis is an ordered cell death process that occurs in physiological and pathological conditions. A disruption of this delicate balance can lead to the development of cancer [27]. Now little is known about the effect of miR-30b on apoptosis in cancer. The controversial expression of miR-30b suggests the complexity of the function of miR-30b. Recently, Li et al [41] found that miR-30b was significantly reduced in response to the oxidative stress stimulation, and miR-30b could inhibit mitochondrial fission and consequent apoptosis. On the contrary, it has been shown that miR-30 can inhibit the self-renewal and induce apoptosis of breast tumor-initiating cells (BT-ICs) by silencing Ubc9 and ITGB3 [42]. Above evidences indicate that miR-30 is a multifunction gene which can inhibit or induce the apoptosis. In current report, we found ectopic expression of miR-30b could induce the apoptosis of gastric cancer cells in vitro, and overexpression of miR-30b could significantly inhibit tumor growth in nude mouse xenograft model by inducing the apoptosis of gastric cancer cells in vivo. Above findings suggest that miR-30b may play the potential role in gastric cancer development by promoting apoptosis of cancer.

To explore the molecular mechanism underlying miR-30b function, it is important to identify its target gene. Recently, several novel targets of miR-30b have been confirmed including p53 [43], Delta-like 4 [44], and Snail1 [45]. In our study, PAI-1was identified as a target gene of miR-30b in gastric cancer. PAI-1 is the principal inhibitor of tPA and uPA, and PAI-1 can blocks the activation of plasminogen. It has been well documented that the expression of PAI-1 is higher in gastric cancer than in control tissues, and PAI-1 may serve as a new prognostic factor in gastric cancer, predicting shorter survival [28], [46], [47]. Interestingly, recent evidence shows the protumorigenic activity of PAI-1 may be through an antiapoptotic function [22]. Our study showed that miR-30b expression was inversely correlated with PAI-1 expression in gastric cancer cell lines and tumor tissues, PAI-1 overexpression could counteract the effect of promoting apoptosis by miR-30b, and ectopic expression of miR-30b had similar promoting-apoptosis effect compared with silencing PAI-1 expression. It suggests that the miR-30b-PAI-1 axis may be involved in the development of gastric cancer. However the downstream pathway is needed to be further investigated.

The development of gastric cancer is characterized by multi-factorial and multistage process, and *H. pylori* has been shown to the leading cause of gastric cancer. Recent reports of our research and other studies have highlighted the regulatory role of miRNAs in *H. pylori* infection and associated diseases. miRNA is a potential bridge from *H. pylori* infection to chronic gastritis and gastric cancer [16], [48], [49]. Regarding to miR-30b, it may have multi-function in *H. pylori* infection and *H. pylori*-associated diseases. Our previous report found miR-30b was able to regulate the autophagy process by targeting ATG12 and BECN1 during *H. pylori* persist infection [19]. Therefore, we hypothesized that miR-30b may play the versatility role in *H. pylori* infection and gastric cancer, however, it needs to be further investigated.

In summary, we report the down-regulation of miR-30b in gastric cancer, and investigate the potential role of miR-30b in tumorigenesis by regulating apoptosis. This study provides new insights into the role of miR-30b in gastric cancers. miR-30b may function as a potential tumor suppressor gene in gastric cancer, and have the potential application as a biomarker or therapeutic target in gastric cancer therapy.
